# Use of Advanced Hybrid Closed-Loop System during Pregnancy: Strengths and Limitations of Achieving a Tight Glycemic Control

**DOI:** 10.3390/jcm13051441

**Published:** 2024-03-01

**Authors:** Parthena Giannoulaki, Evangelia Kotzakioulafi, Alexandros Nakas, Zisis Kontoninas, Polykarpos Evripidou, Triantafyllos Didangelos

**Affiliations:** 1 Department of Clinical Nutrition, University General Hospital of Thessaloniki AHEPA, 54636 Thessaloniki, Greece; 2 Diabetes Center, 1st Propaedeutic Department of Internal Medicine, Faculty of Medicine, University General Hospital of Thessaloniki AHEPA, Aristotle University of Thessaloniki, 54636 Thessaloniki, Greece; ekotzaki@auth.gr (E.K.); al.nakas@hotmail.com (A.N.); drziko2401@gmail.com (Z.K.); didang@auth.gr (T.D.)

**Keywords:** advanced hybrid closed-loop system, diabetes mellitus type 1, glycemic control, pregnancy

## Abstract

**Background**: Pregnant women with type 1 diabetes mellitus (T1DM) face an elevated risk of complications for both themselves and their newborns. Experts recommend strict glycemic control. The advanced hybrid closed-loop (AHCL) system, though not officially approved for pregnant T1DM patients, is promising for optimal glycemic control. **Methods**: We collected CGM metrics, HbA1c levels, insulin pump settings, and doses from a 33-year-old pregnant woman with 23-year history of T1DM from the 6th week of gestation to birth. She was initially on continuous insulin pump therapy with CGM and switched to the AHCL system (MiniMed^TM^ 780G, Medtronic, Northridge, CA, USA) between weeks 13 and 14. **Results**: The AHCL system improved glycemic control from weeks 14 to 26, achieving international guidelines with TIR = 72%, TAR = 24%, TBR = 4%. At week 30, TIR was 66%, TAR 31%. By altering diet and adding ‘fake carbohydrates’, she maintained TIR ≥ 70%, TBR ≤ 4%, TAR ≤ 26% from week 34 to birth. A healthy 4 kg, 53 cm baby boy was born at week 38. **Conclusions**: The use of the AHCL system holds significant promise for improving glycemic control in pregnancy. Optimal glycemic control with MiniMed^TM^ 780G in pregnancy requires accurate carbohydrate counting, specific timing of insulin doses in relation to meal consumption and dietary choices that reduce the glycemic load of meals continue to be crucial factors in achieving optimal glycemic control during pregnancy using the MiniMedTM 780G system. Further research and clinical studies are needed to explore the full potential of these advanced systems in managing T1DM during pregnancy and optimizing maternal and neonatal outcomes.

## 1. Introduction

In recent literature, it has been established that the presence of type 1 diabetes mellitus (T1DM) during pregnancy poses an increased risk of complications for both the mother and the neonate [1]. Insulin requirements typically increase by two to three times during the second and third trimesters, with significant day-to-day variability, emphasizing the need for dose adjustments. The primary goal during pregnancy is to increase the time in range (TIR) safely and quickly, while reducing time above range (TAR) and glycemic variability [2,3]. The international consensus on glycemic targets in pregnancy has recommended stringent blood glucose targets, aiming for time spent within 63–140 mg/dL more than 70%, with less than 4% falling below 63 mg/dL and less than 25% exceeding 140 mg/dL [4]. Achieving these targets without inducing severe hypoglycemia presents a significant challenge.

Existing studies, such as the CONCEPTT study [5] and a Swedish cohort [6], have revealed suboptimal daily glycemic control among women with T1DM during pregnancy, despite the utilization of continuous glucose monitoring systems and insulin pumps. Furthermore, these studies have reported persistently high rates of excessive fetal weight for gestational age. Consequently, there is a pressing need for novel treatment approaches in the management of T1DM during pregnancy that offer optimal glycemic control.

The MiniMed^TM^ 780G system, an automated insulin delivery system currently not officially approved for use during pregnancy, is the sole system of its kind available in the country. It incorporates an advanced hybrid closed-loop (AHCL) system algorithm, an innovation derived from the MiniMed 670G system, enabling automatic basal rate adjustment and auto-correction doses. The AHCL algorithm includes automatic insulin delivery every 5 min, with 3 selectable adjustable glucose targets of 100, 110 and 120 mg/dL, with automatic bolus correction every 5 min. It requires meals (carbohydrate amount) to be announced by the user to achieve optimal glycemic results. Administration of automatic correction every 5 min improves glycaemia throughout the day, mitigates potential inaccuracy of carbohydrate amount calculation, late or no meal bolus administration, and corrects daily glucose variability without user intervention. In addition, the system calculates the daily insulin dose independently in order to determine the correction ratio. The patient can only alter the insulin to carb ratios and active insulin time in this system. Finally, the exit conditions from auto mode have been changed from those used in the 670G algorithm, reducing the burden on the user while ensuring safety [7]. The Guardian 4 sensor used in the system no longer needs calibration.

In real-world settings [7], a majority of users of this system achieve a time in range (TIR) (70–180 mg/dL) greater than 70% and a glucose management indicator (GMI) less than 7%, while experiencing minimal hypoglycemic events. These promising outcomes make the system a potential therapy for deployment during pregnancy.

The use of hybrid closed-loop insulin delivery has shown promise in improving glycemic control among pregnant women with T1DM in pilot studies [8,9,10,11]. Despite the limitations in study design, such as small sample sizes and crossover designs, some studies have reported increased TIR and reduced hypoglycemia. While the available evidence regarding the use of investigational closed-loop insulin delivery systems during pregnancy is promising, data and guidance are still needed regarding the impact of different systems on glycemic control in pregnant T1DM women.

Our review of the current literature identified two published case reports [12,13] and a case series [14] using the Minimed Medtronic 670G hybrid closed system in pregnant women with T1DM. To the best of our knowledge, no case report exists in the literature that uses the MiniMed^TM^ 780G system in women with T1DM during gestation. Moreover, five small observational retrospective studies [15,16,17,18,19] examined the impact of a HCL system on the glycemic control of pregnant women with T1DM and maternal-neonatal complications. Four studies evaluate the use of the MiniMedTM 780G [15,16,17,18] system and one the use of Control-IQ system [19].

In this paper, we present a case study examining the experience of a pregnant woman with T1DM who used the MiniMed^TM^ 780G system during the latter part of the first trimester and postpartum, despite lacking official approval for use during pregnancy.

## 2. Materials and Methods

We gathered data on continuous glucose monitoring metrics, hemoglobin A1c (HbA1c) values, insulin pump settings, and insulin doses from a 33-year-old woman with T1DM, who was an outpatient at our Diabetes Center. She had been diagnosed with T1DM for 23 years and did not have any microvascular complications or comorbidities. She followed a vegetarian diet, with a body weight of 52 kg and a body mass index of 19 kg/m^2^. The patient had an unplanned pregnancy, which was confirmed during the 6th week of gestation. As soon as the patient found out about the pregnancy, she visited the Diabetes Center. She was switched to the hybrid closed-loop insulin delivery system between the 13th and 14th week of pregnancy.

## 3. Results

She was initially on continuous insulin pump therapy (Minimed^TM^ 640G) along with a Continuous Glucose Monitoring (CGM) system with Predictive Low Glucose Suspend (PLGS) technology, that allows the suspension of insulin delivery when hypoglycemia is predicted by the algorithm. Her HbA1c level was 6.6%, and the mean sensor glucose level was 136 ± 38 mg/dL with a coefficient of variation of 27.8%. The time in range (TIR) of 63–140 mg/dL was 50%, time below range 63 mg/dL (TBR) was 4%, and time above range 140 mg/dL (TAR) was 46%. Before pregnancy, her HbA1c value was 7.4%. The patient’s pump settings at the beginning of pregnancy are presented in Table 1.

However, her glycemic control did not align with international recommendations for pregnancy. After evaluating the patient’s daily glycemic profile reports, which showed postprandial excursions between 12:00–16:00, adjustments were made to the ICR during that time. The revised ICRs were as follows: 00:00–10:00 = 18 g; 10:00–12:00 = 20 g; 12:00–14:00 = 18 g; 14:00–16:00 = 16 g; 16:00–00:00 = 18 g.

To optimize glycemic control and meet the nutritional needs of the first trimester of pregnancy, an individualized diet was established. The diet provided 1800 calories and covered the daily reference intakes for carbohydrate (175 g), protein (71 g), and fiber (28 g) [20]. Caloric adjustments were made throughout pregnancy in each trimester. The diet was regularly reviewed by a registered dietitian specialized in T1DM and adjusted based on glycemic control during follow-up (Table 2). Her mean meal frequency per day was 5 and varied from 4–6 meals depending on her symptoms.

At the eighth week of gestation, the patient’s TIR was 47%, TBR was 8%, TAR was 45%, and the mean sensor glucose level was 137 mg/dL. In order to reduce hypoglycemia and rebound hyperglycemia, the Suspend Before Low threshold was increased to 70 mg/dL, and the 24 h glucose target was reduced to 100 mg/dL. The AIT was shortened to 3 h. Additionally, due to increased insulin resistance in the morning, the ICR for the morning meal was decreased, and at that time it was reduced by 2 g.

At the 12th week of gestation, the patient experienced intense hyperemesis. During this period, her TIR was 49%, TBR was 14%, TAR was 37%, and the mean glucose level was 126 mg/dL with a coefficient of variation of 39%. To mitigate time spent in hypoglycemia, a recommendation was made to increase ICRs by 3 g throughout the 24 h period.

Considering the challenges in achieving optimal glycemic control during the early stages of gestation, the multidisciplinary team discussed transitioning the patient to the AHCL system. With the patient’s consent, she was switched to the Hybrid Closed-Loop insulin delivery system (MiniMed^TM^ 780G) between the 13th and 14th weeks of pregnancy. The patient’s glycemic control data before using the 780G system are presented in Table 3.

**Figure 1:**
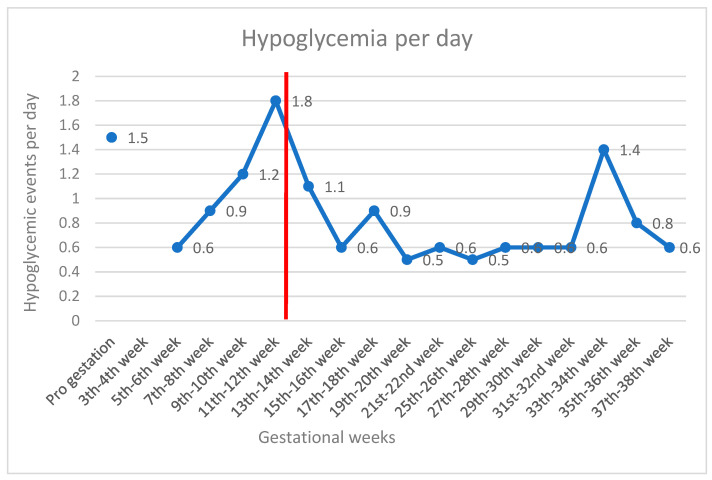
Hypoglycemic events per day by gestational weeks. The red line indicates the switch to AHCL. 1st to 4th week missing data because she did not wear any sensor.

The utilization of the AHCL system resulted in improved glycemic control from the 14th to the 26th week (3 months of using the system), with the achievement of international recommendations for time targets within a specific range of blood glucose values during pregnancy. During this period, the patient’s TIR (63–140 mg/dL) was 72%, TAR was 24%, and TBR was 4% (<54 g/dL 2%). Sensor wear was 95% with SmartGuard use 96% and mean sensor glucose at 118 mg/dL with CV 28.2.%. Hypoglycemia per day was recorded and is presented in Figure 1 throughout the pregnancy.

Additionally, the data presented in Table 4 provide information on HbA1c levels and the insulin pump settings, as well as the insulin doses at the beginning of pregnancy and at the end of each trimester. Moreover, Table 3 provides the CGM reports’ metrics during each trimester.

Despite achieving the goals of glycemic control in pregnancy during the second and third trimester using the 780G system, several challenges were encountered during the third trimester. At the 30th week of gestation, analysis of the CGM report revealed a decrease in time in range (TIR = 67%) and an increase in Time Above Range (TAR = 30%). Based on these findings, a recommendation was made to reduce all ICRs by 2 g due to observed postprandial hyperglycemia.

However, contrary to expectations, glycemic control deteriorated after one week (TIR = 66%, TAR = 31%). Further examination of daily glycemic profiles revealed that the system had activated the “safe meal bolus” function, resulting in a lower insulin dose being administered to avoid postprandial hypoglycemia, rather than using the ICR setting at that time. To address this issue, we thought “out of the box” and recommended to restore the ICRs to their previous setting, increasing them to disable the “safe bolus” function, and to address postprandial excursions through nutritional adjustments. This involved adding animal protein and high-quality fats to meals.

After one week, there was a slight improvement in TIR (67%), but it did not reach the target of >70%. Therefore, at the 32nd week, considering the late gestation stage and limited time frame, we decided to “trick” the algorithm by introducing extra “fake carbohydrates” during main meals in order to increase the meal bolus doses. The recommendation involved adding an additional amount of “fake carbohydrates” (+10 g at breakfast and dinner, +20 g at lunch) to the actual amount of carbohydrates consumed by the patient. Furthermore, it was advised to consume the meal 30 min after the bolus dose. As a result, from the 34th week of gestation until the birth of the newborn at the 38th week, glycemic targets were achieved with TIR ≥ 70%, TBR ≤ 4%, and TAR ≤ 26%.

Consequently, a healthy baby boy weighing 4 kg and measuring 53 cm in height was born via a planned caesarean section at the 38th week of gestation. The patient continued to use the AHCL system post-pregnancy.

During the follow-up of the pregnancy, it was of utter importance to the team to record the patient’s view on the benefits and burdens of the specific system. Her main belief was that, in general, the system had succeeded in achieving the glucose targets based on the international recommendations for pregnancy and in preventing hypoglycemia better, compared to the previous system. However, there was a period during the last trimester that the system failed to control glucose levels due to rapid hormonal changes (Table 5).

## 4. Discussion

The presence of T1DM during pregnancy is associated with an increased risk of maternal and neonatal complications. Women with T1DM have a 2–5 times higher risk of adverse outcomes during pregnancy, including congenital anomalies, stillbirth, and perinatal mortality [1].

During pregnancy, women with T1DM face specific challenges related to glycemic control. In the first trimester, there is an increased risk of hypoglycemia, and women may experience reduced awareness of hypoglycemia due to altered insulin needs. Additionally, there is a risk of diabetic ketoacidosis occurring at lower blood glucose levels [20].

According to the literature, a significant delay of 30 min in postprandial glucose disposal is observed, as pregnancy progresses. The delay between the postprandial maximum blood glucose concentration and the glucose disposal is 45 min in the early gestation and 75 min in the late gestation. The factors that are attributed to that are the delayed attainment of maximum blood postprandial insulin concentration and increased peripheral insulin resistance. Consequently, tissue glucose disposal is impaired, resulting in prolonged postprandial hyperglycemia in late pregnancy. Therefore, optimal administration of mealtime insulin bolus is time-dependent, with recommended intervals of 15 min in early pregnancy, and 30–40 min in late pregnancy before a meal [21]. In our case, this pathophysiological mechanism revealed the need to delay meal consumption for 30 min after the bolus dose, during the third trimester.

Scientific evidence demonstrates that even small changes in maternal blood glucose levels have a substantial impact on maternal and neonatal outcomes. Women with HbA1c levels exceeding 6.1% after 24 weeks of gestation have significantly higher rates of preterm delivery, newborn large for gestational age (LGA), ICU admissions, and a threefold increased risk of premature death [22]. These findings have led to the establishment of international recommendations for stringent glycemic targets in pregnancy for individuals with T1DM, with a target time in range (TIR) of 63–140 mg/dL exceeding 70% throughout pregnancy [4]. In the present report, HbA1c was 6.2% during and 6.1% after the 24th week of gestation using the AHCL system, which was initiated in the 14th week of gestation.

In the current literature, the use of advanced therapeutic technologies, such as continuous subcutaneous insulin pumps and CGM systems, in achieving these strict glycemic targets during pregnancy has yielded scarce results. Studies evaluating the use of insulin pump systems with CGM during pregnancy have not consistently demonstrated significant differences in glycemic parameters in the third trimester, irrespective of the timing of pump initiation (before conception or during pregnancy). However, initiating an insulin pump system with CGM before conception appears to be more effective in achieving the recommended glycemic targets during the first two trimesters of pregnancy in women with T1DM [5,23]. In our case report, even though the pregnant female was on sensor augmented pump therapy before conception, she could not meet the glucose targets for pregnancy during the first trimester.

The available literature suggests that while advanced technology improves glycemic control in women with T1DM during pregnancy, it often falls short of achieving optimal outcomes according to international recommendations [5,6]. Real-life data [22] indicate that only a small percentage of women with T1DM in pregnancy, around 15–16%, achieve the target HbA1c level of 6.5%. This highlights the need for specific closed-system insulin delivery algorithms designed for pregnancy, such as AHCL.

In the presented case, the use of the MiniMed^TM^ 780G system provided improved glycemic control during the second and third trimesters of pregnancy, aligning with the international recommendations for TIR, TAR and TBR in pregnancy. However, challenges were encountered during the third trimester, leading to the need for further adjustments in the therapeutic approach. Strategies such as modifying ICRs, addressing postprandial hyperglycemia through nutritional adjustments, “tricking” the algorithm by introducing “fake” carbohydrates and weekly review of the patient’s glycemic profiles were employed to optimize glycemic control. These interventions resulted in improved glycemic outcomes until the birth of a healthy baby boy at the 38th week of gestation.

However, failure to achieve glycemic targets from the beginning of pregnancy (1st trimester) resulted in the inability to prevent macrosomia. This aligns with a retrospective analysis from Scott et al. [24], who demonstrated that maternal glucose trajectory, which is achieved in 10 weeks of gestation, determines the association of birth weight for the rest of the pregnancy. Additionally, normal birth weight is associated with mean sensor glucose ≤126 mg/dL in 10 gestation weeks with TIR = 55–60% from the 8th to the 10th gestation week, with the target set to 70% in the following weeks. Our patient had mean glucose 129 mg/dL and 50% TIR in the 10th week of gestation, which may justify the LGA.

In terms of overall satisfaction by using this particular HCL system, our case reported high. This belief as well as the documented advantages, weaknesses and suggested improvements of the system are in accordance with the results of the qualitative content analysis of Munda et al. [18]. The main advantage of using the HCL system was the easier achievement of the strict target of time in the glucose range with lower time below glucose range and hypoglycemic episodes during gestation. On the other hand, the main weakness was the slow response of the algorithm to the postprandial glucose excursions, especially during the third trimester. It is important to note that the system has certain limitations in terms of its algorithm. The most stringent available glucose target for basal rate is 100 mg/dL, while the target for auto corrections is set at 120 mg/dL. This does not meet the fasting blood glucose level of <95 mg/dL, that is the glucose target for pregnancy [20]. Additionally, the system does not appear to have the capability to make rapid adjustments in insulin administration as required during pregnancy, due to the major hormonal changes. Specifically, around 16 weeks, insulin resistance begins to increase, and total daily insulin doses increase linearly 5% per week through the 36th week [20]. In MiniMed^TM^ 780G the algorithm adjusts the basal rate by taking account of the sensor glucose values of the last 7 days. Moreover, the main suggested improvement by the women, both in our report and Munda et al. study [18], was the faster adaptation of the algorithm to changes in insulin needs.

Pilot studies on the use of automated insulin delivery systems in pregnancy have yielded promising results [8,9,10,11]. However, it is important to note that different systems may suit different users, and adjustments are often required in terms of system aggressiveness and the time administration of meal bolus doses. Regular assessment of glycemic profile reports on a weekly basis remains crucial.

To our knowledge, there are four small observational real-life retrospective single-center studies [15,16,18,19] and one observational multicenter retrospective study [17] which examined the impact on glycemic profile, maternal and neonatal complications in pregnant women with T1DM using commercially available automated insulin delivery (AID) systems. An observational multicenter retrospective analysis [17] of thirteen pregnant women using the MiniMed^TM^ 780G system including a twin pregnancy, showed improvement in glycemic control and met the recommended glucose targets only during nighttime (TIR > 70%). However, maternal and fetal complications remained high. Similar results were also described by Albert et al. [15] who evaluated six pregnant women using MiniMed^TM^ 780G system and reported challenges in achieving the recommended glucose targets, mean TIR was 67%, 65.5% and 65.5% in the first, second and third trimesters of gestation, respectively. Importantly, two serious adverse fetal events were observed. On the other hand, Munda et al. [18] reported six women treated with the same HCL system and revealed high mean TIR values during the second and third trimester (78.6% and 83.6%, respectively) with mean TBR > 4% during the first and second trimester. The HbA1c value remained at ≤6.0% (42 mmol/mol) throughout pregnancy, but perinatal outcomes were not documented. Furthermore, Dodesini et al. [16] examined eight pregnant women with T1DM and the impact on metabolic control, evaluated by CGM metrics using MiniMed^TM^ 780G during gestation. Our findings are in accordance with this study [16], due to glycemic control during the second and third trimester but not during the first trimester. Our case experienced more TBR during the 1st trimester resulting in a lower TIR. The late initiation of the AHCL system (14th week of gestation) may contribute to this outcome. Finally, Wang et al. reported four cases using the AID Control-IQ system during pregnancy. All participants achieved a mean TIR value > 70%, with three out of the four reaching HbA1c ≤ 6.1% (43 mmol/mol) by the third trimester. Despite these positive outcomes, three neonates were born LGA, one woman developed pre-eclampsia, and three women underwent cesarean section births. An ongoing randomized controlled trial comparing the Minimed 780G system to standard care for T1DM in pregnancy is expected to provide more comprehensive insights [25].

This is the first time in our clinical experience with technology in T1DM management since 2001 [26] where we observed achievement of strict glycemic control through pregnancy without serious complications and hypoglycemic events. From a clinical perspective, utilizing a specific AHCL system, such as the MiniMed^TM^ 780G system, strict glycemic targets in pregnancy with T1DM were feasible. Based on patient feedback, it appears that the AHCL system has been a safe and successful option, resulting in increased patient satisfaction and a potential reduction in psychological stress. However, this algorithm showed notable limitations in achieving the optimal glycemic control in women with T1DM during the third trimester of pregnancy due to the rapid changes in insulin needs that occur in this period time and so, there is a need for close medical and dietary monitoring by the multidisciplinary health-care team.

## 5. Conclusions

In conclusion, the use of AHCL system holds significant promise for improving glycemic control in pregnancy. As technological developments continue to progress and become more efficient, it is reasonable to expect better glycemic control and improved outcomes in pregnancy in the future. Accurate calculation of meal carbohydrates, timing of insulin doses in relation to meal consumption, and dietary choices that reduce the glycemic load of meals continue to be crucial factors in achieving optimal glycemic control during pregnancy using the MiniMedTM 780G system. Further research and clinical studies are needed to explore the full potential of these advanced systems in managing T1DM during pregnancy and optimizing maternal and neonatal outcomes.

## Figures and Tables

**Table 1:** Patient’s pump settings at the beginning of pregnancy.

Time	ICR (g)	ISF (mg/dL)	Glucose Target (mg/dL)	AIT (h)	Suspend before Low (mg/dL)
0.00	18	60	100–120	4	60
10.00	20	60	100–120	4	60
14.00	18	60	100–120	4	60

ICR = Insulin-to-Carb Ratio; ISF = Insulin Sensitivity Factor; AIT = Active Insulin Time.

**Table 2:** Body weight status and individual’s reported intake.

	Weight (kg)	BMI (kg/m^2^)	Kcal	Carbs (g)	Protein (g)	Fat (g)	Fiber (g)
Pro gestation	52	19.1	1800	157	71	90	26
5th–6th week	52.2	19.2	1800	165	78	92	24
7th–8th week	53.3	19.6	1800	173	72	94	27
12th–13th week	54.4	20	2000	178	85	99	25
18th–19th week	57.8	21.2	2000	180	95	101	28
23rd–24th week	59.4	21.8	2000	190	85	99	22
28th–29th week	62	22.8	2000	187	88	99	26
32nd–33rd week	65	23.87	2200	180	110	115	27
34th–35th week	67	24.6	2200	191	110	109	25

BMI: Body Mass Index, Kcal represent the proposed meal plan according to nutritional demands from the dietitian and macronutrient distribution is the actual consumption by the patient.

**Table 3:** Data of glycemic control throughout the pregnancy.

Trimester	Mean SG (mg/dL)	CV (%)	63–140 mg/dL (%)	<63 mg/dL (%)	<54 mg/dL (%)	>140 mg/dL (%)	Sensor Wear (%)	Smartguard Use (%)
Beginning of pregnancy	136	27.8	50	4	0	46	74	0
First	126	39	49	15	6	37	91	0
Second *	121	28.3	72	2	1	26	97	92.5 *
Third	117	30.2	70	4	1	26	97	96.7

SG = Sensor Glucose; CV = Coefficient of Variance. * AHCL was initiated in 13th gestational week.

**Table 4:** HbA1c levels, insulin pump settings and insulin doses at the beginning of pregnancy and at the end of each pregnancy trimester.

	Beginning of Pregnancy (5–6th Week)	1st Trimester (11–12th Week)	2nd Trimester (23–24th Week)	3rd Trimester (37–38th Week)
HbA1c (%)	6.6	6.3	6.2	6.1
ICR (g)	18–20	16–20	7–12	7–10
ISF (mg/dL)	60	60	80	60
Basal (units/day)/(%)	15.1 (74%)	14.2 (58%)	7.6 (28%)	15.5 (36%)
Bolus (units/day)/(%)	5.3 (26%)	10.2 (42%)	19.6 (72%)	27.9 (64%)
TDD (units/day)	20.4	24.4	27.2	45.6

HbA1c = Glycated Hemoglobin; ICR = Insulin-to-Carb Ratio; ISF = Insulin Sensitivity Factor; Bolus = Insulin Doses (meal + correctional); TDD = Total Daily Dose.

**Table 5:** Patient’s opinion on the use of the advanced hybrid closed-loop system.

Advantages	Disadvantages	Feedback
“In general, the system has done a great job during the entire pregnancy, even though it is not programmed for pregnancy.”	“The only period I felt like the pump “is failing me” was during the last trimester when the hormonal changes were extreme…”	“Due to constant changes in insulin needs during pregnancy two factors are crucial
“…the system is way better compared to my former regimen…”	“…it takes a while to lower a high glucose result and “fake” carbs are used as a strategy, whereas with my previous system you can intervene directly and correct it.”	1. The medical team to review my data weekly or sooner and act immediately
“…it does a great job at preventing hypoglycemia.”		2. The system to be able to change the basal rates within 1–2 days and not wait longer.”

## Data Availability

The data that support the findings of this study are not available due to privacy restrictions.
